# PGsim: A Comprehensive and Highly Customizable Personal Genome Simulator

**DOI:** 10.3389/fbioe.2020.00028

**Published:** 2020-01-28

**Authors:** Liran Juan, Yongtian Wang, Jingyi Jiang, Qi Yang, Qinghua Jiang, Yadong Wang

**Affiliations:** ^1^School of Life Science and Technology, Harbin Institute of Technology, Harbin, China; ^2^School of Computer Science and Technology, Harbin Institute of Technology, Harbin, China

**Keywords:** personal genome, genome simulation, variant simulation, genome variation, computational tools

## Abstract

Although genome sequencing has become increasingly popular, the simulation of individual genomes is still important. This is because sequencing a large number of individual genomes is costly and genome data with extreme and boundary conditions, such as fatal genetic defects, are difficult to obtain. Privacy and legal barriers also prevent many applications of real data. Large sequencing projects in recent years have provided a deeper understanding of the human genome. However, there is a lack of tools to leverage known data to simulate personal genomes as real as possible. Here, we designed and developed PGsim, a comprehensive and highly customizable individual genome simulator, that fully uses existing knowledge, such as variant allele frequencies in global or world main populations, mutation probability differences between protein-coding regions and non-coding regions, transition/transversion (Ti/Tv) ratios, Indel incidence, Indel length distribution, structural variation sites, and pathogenic mutation sites. Users can flexibly control the proportion and quantity of known variants, common variants, novel variants in both coding and non-coding regions, and special variants through detailed parameter settings. To ensure that the simulated personal genome has sufficient randomness, PGsim makes the generated variants more real and reliable in terms of variant distribution, proportion, and population characteristics. PGsim is able to employ a huge volume database as background data to simulate personal genomes and does not require SQL database support. Users can easily change the variant databases used as needed. As a Perl script, there is no obstacle to running PGsim on any version of the MAC OS or Linux systems, and no libraries, packages, interpreters, compilers, or other dependencies need to be installed in advance. The PGsim tool is publicly available at https://github.com/lrjuan/PGsim.

## Introduction

Although personal genome sequencing has become increasingly popular, the simulation of individual genomes remains important. Sequencing a large number of individual genomes remains a costly procedure. Several international projects have sequenced genomes on a large scale. However, many complex diseases lack sufficient whole-genome sequencing samples. Some typical familial inherited diseases are also unable to be sequenced using the new technologies.

High-throughput sequencing technology still has several disadvantages in terms of individual genome sequencing. For example, it is difficult to obtain large structural variations (SV) and extreme examples and boundary conditions. Fatal genetic defects often lead to miscarriage and stillbirth and thus cannot be sequenced. There are also privacy and legal barriers to the application of many real data.

On the other hand, the large sequencing projects of recent years have provided a deeper understanding of the human genome, facilitating the simulation of individual genome data. In addition to the known basic genetic laws and general human genome sequences, the advancement of large projects, such as the 1000 genomes project (KGP) (Sudmant et al., 2015), have made the human variation databases more complete. Representative individuals of different populations have been determined. A large number of genome-wide analysis studies (GWASs) have identified millions of disease-related variants. Databases such as ClinVar (Landrum et al., 2015) and HGMD (Stenson et al., 2012) have been established. Fully leveraging these data and information, individual genome data that meet a variety of research needs can be simulated.

Hundreds of genome simulation methods and tools have been developed (Peng et al., 2018), which can be divided into three broad groups: (1) coalescent simulators for population genomes evolving under particular evolutionary models (Carvajal-Rodríguez, 2008), such as GENOME (Liang et al., 2007), GeneEvolve (Tahmasbi and Keller, 2017), and SFS_CODE (Uricchio et al., 2015); (2) simulation tools for case–control GWAS data, such as simGWA (Yang and Gu, 2013), simGWAS (Fortune and Wallace, 2018), GWAsimulator (Li and Li, 2007), and TraidSim (Shi et al., 2018); and (3) simulators for various types of genome variants and sequences, such as FIGG, simuG, VST, VarSim, Xome-Blender, and SVEngine. FIGG generates large numbers of whole genomes with known sequence characteristics based on the direct sampling of experimentally known or theorized variations (Killcoyne and del Sol, 2014). simuG simulates SNPs, Indels, CNVs, Inversions, and Translocations for different organisms (Yue and Liti, 2019). Simulome can also simulate genome sequences and variants for prokaryotic pseudo-genomes (Price and Gibas, 2017). VST provides a forward-time simulation engine that simulates real nucleotide sequences of the human genome using DNA mutation models (Peng, 2015). VarSim synthesizes diploid genomes with germline and somatic mutations based on a realistic model for assessing alignment- and variant-calling accuracy in high-throughput genome sequencing tools (Mu et al., 2014). SVEngine simulates structural variants *in silico* and provides variants with different allelic fractions and haplotypes (Xia et al., 2018). Xome-Blender generates synthetic cancer genomes with user-defined features such as the number of subclones, the number of somatic variants, and the presence of copy number alterations (Semeraro et al., 2018). Sim1000G is an R package for simulating variants in genomic regions among unrelated individuals or families using KGP data (Dimitromanolakis et al., 2019). Finally, a logistic regression method has been developed for simulating realistic genomic data with rare variants based on the KGP data (Xu et al., 2013).

In recent years, a large number of individual genomes have been sequenced. Genomics and population genetics studies benefit from advanced technology and further deepen the understanding of the human genome. Knowledge such as variant allele frequencies in global or world main populations, mutation probability differences between protein-coding regions and non-coding regions, transition/transversion (Ti/Tv) ratios, SV sites, and pathogenic mutation sites have been continuously investigated. Existing tools are not yet able to take full advantage of such rapidly increasing genomic knowledge in individual genome simulations, however.

In this paper, we developed PGsim, a comprehensive and highly customizable personal genome simulator, by integrating the above information. By setting parameters, users can flexibly simulate individual genomes. Options such as variant number, variant type, and percentages of variants generated from different sources can be configured. Users can also incorporate SVs and disease-related variants in the simulated personal genome based on known SV databases and disease-related variant databases. The numbers of these two types of variants can be set on demand.

PGsim first plans an individual genome simulation by a series of parameters specified by the user and automatically analyses the multiple databases that are required for the simulation. Then, according to the planning results, PGsim extracts known variants that meet the user’s needs from the databases and randomly generates new mutations that comply with mutation laws. PGsim ensures that there is no overlap or conflict between variants from different sources, and the user-requested number of variants is guaranteed. Finally, PGsim generates diploid personal genome sequences and other auxiliary information files based on the simulated personal genome variants and the reference genome. The simulator requires variant databases in VCF format, and Bgzip/Tabix compressed/indexed VCF files are recommended.

In the following section, we first describe the overall workflow of PGsim. Then, the specific strategy for each step in the personal genome simulation is introduced. Databases are analyzed next, followed, sequentially, by a discussion of parameter configurations and an explanation of implementation methods. Finally, the time consumption of PGsim with typical parameters is studied.

## Materials and Methods

### Overall Workflow

**FIGURE 1:**
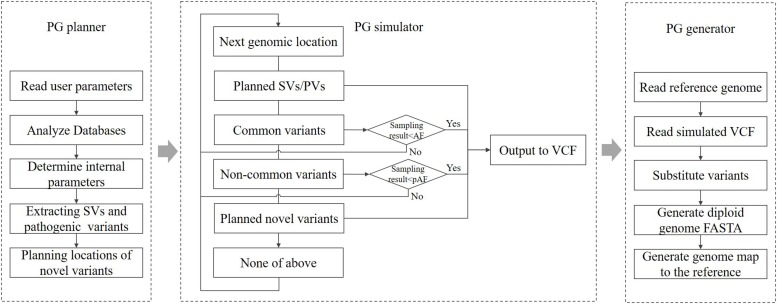
The overall workflow of PGsim.

According to the parameters specified by the user, PGsim comprehensively considers type, source, location, allele frequency (AF), Ti/Tv ratio, and other information about genomic variants and randomly extracts them from known variant databases or generates them randomly based on the user configuration. As shown in Figure 1, PGsim consists of three components: (1) personal genome planner (PG_planner), (2) personal genome simulator (PG_simulator), and (3) personal genome generator (PG_generator).

#### Personal Genome Planner

Personal genome planner plans the personal genome simulation. It reads the detailed parameter settings input by the user, analyses the variant databases, and calculates several internal parameters based on the input parameters and the database analysis results.

For known and common variants in the databases, we calculate an AF correction factor so that, subsequently, these variants can be selected in a manner that is relatively consistent with their AF in the background population. For rare or unknown variants, a series of genomic coordinates are randomly generated as mutation positions. The generated positions are prevented from falling into unmeasured regions near the telomeres or centromeres. Moreover, considering the different mutation laws between protein-coding regions and non-coding regions, PG_planner allows users to set a specific number of coding region mutations, which is independent from the mutations in the non-coding region. PG_planner also randomly extracts the user-specified numbers of structural variants and disease-related variants.

#### Personal Genome Simulator

Personal genome simulator simulates individual genome variants based on the planning scheme output by PG_planner. PG_simulator merges variants simulated from different sources, including ones randomly selected from known variant databases and ones randomly generated by PG_planner. The simulated variants are output in VCF format, and their genotypes are also determined.

For known variants in the databases, the PG simulator will sample them one by one according to the AF of the background population. When the random number obtained from the sampling of a variant is less than its corrected AF, the variant will be selected; otherwise, it will be skipped. Each autosomal variant or the X chromosomal variant of female individuals will be sampled twice as the genotype on the diploid genome.

For newly generated variants, PG_simulator further determines the length, type, and other information based on the mutation laws. The features of the variants, including the Ti/Tv ratio, Indel rate, and frame shifting rate of coding variants, are set by the input parameters, while the necessary randomness is kept. The novel variants are all assigned as heterozygous variants on a random haploid genome.

Structural and disease-related variants can only be extracted from known databases and added directly to the generated personal genome. They are also heterozygous.

#### Personal Genome Generator

Personal genome generator produces the diploid sequence of the simulated individual genome based on the reference genome data and the individual genome variants generated by PG_simulator. PG_generator loads the data from both variants data and the reference genome, and then replaces the base of the reference genome sequence at the mutation position with the mutated base, including Indels and SNVs. The result is output in FASTA format. PG_generator produces a FASTA file for each haploid.

Because most known SVs lack the necessary, detailed sequence-level information, the PG_generator will ignore the SVs in the input personal genome variant data. The PG_generator will also generate a set of coordinate mapping data for comparing the coordinates between both haploid genome sequences and the reference genome sequence. Such coordinate differences are caused by Indels.

### Databases

For personal genome simulation, PGsim not only needs to seek authenticity but also should satisfy randomness as much as possible. We used six sequence and variant databases in the individual genome simulation process: (1) reference genome sequences, (2) protein-coding regions, (3) known variant databases, (4) common variant databases, (5) SV databases, and (6) disease-related variant databases (Table 1).

**TABLE 1:** Databases of variant sources.

**Database**	**Format**	**Candidates**
Reference genome	FASTA	GRCh38, GRCh37/hg19…
Coding regions	BED	RefSeq Gene, knownGene, ensemblGene, GENCODE…
All known variants	VCF	dbSNP151, KGP.phase3, ExAC
Common variants	VCF	Common SNP 151, KGP.phase3
Structural variations	VCF	dbVar, DGVa…
Disease-related variants	VCF	Clinvar, HGMD…

PGsim employs these data in the form of files without the need to set up a SQL database. Users can easily select and replace the data files. Among them, the reference genome sequence data are stored in a single file in the FASTA format. To save disk space, PGsim can directly read gzip-compressed FASTA data. The coding region information should be in non-overlapping BED format. Only the first three columns are required. The coding region annotations can be derived from RefSeq, known Gene, GENCODE, etc.

Variant data are stored in VCF format files, including common variant and known variant databases. The common variant database should contain the AF information of the user-specified background population. This information is generally located in the INFO field of the VCF files.

The background population can be the entire human population, a sample cohort of a certain sequencing projects, or a specific population/super population. Users can change the parameters to set the background AF used in the simulation process and then obtain the simulated personal genome that matches the genetic characteristics of the specified population. Not all variants in the common variant database are naturally considered common variants; only those variants with corresponding allele frequencies greater than a certain threshold (default 0.01) will be considered common variants in the specific background population context.

The known variant database should contain all known variations that users expected to occur in the simulated individual genome. Unlike the common variant database, PGsim does not require variants in this database to carry corresponding AF information. All variants have equal probability to be selected.

By default, the known variant database may include common variants, but PGsim only selects non-common variants from it. The common variants will be selected from the common variant database. In the database setting of the parameter configuration, users can set the common variant database and the known variant database to be the same file. In this case, variants with corresponding allele frequencies greater than a threshold value will be considered common variants. The other variants will be considered as non-common variants. We recommend dbSNP and common SNP as the databases of known variants and common variants, respectively. Users can also choose the variant collection from a large sequencing program, such as KGP or GSP, as the background data for known variants.

Considering the complexity of SVs, PGsim does not provide randomly generated SVs. Instead, we only extract SVs from a known SV database. PGsim ensures that the randomly extracted SVs neither overlap with each other nor conflict with other variants simulated from other sources. We recommend dbVar as the SV database.

PGsim extracts the user-specified number of disease-related variants from the disease-related variant database and incorporates them into the simulated personal genome data. Only variants with “Pathogenic” and “Likely pathogenic” clinical significance have the chance to be selected. We recommend that users select ClinVar as the disease-related variant database.

### Variant Simulation

Variants are the key feature of an individual genome. Thus, variant simulation is the essential step of personal genome simulation. PGsim provides various highly customized parameters for realistic, reliable, and flexible simulations of individual genomic variants.

As shown in Table 2, there are two parameters that globally control the simulation process of individual genome variants: gender and overall variation rate (OVR). Gender determines the sex chromosome composition of the simulated individual genome. It can be set to male, female, or any. The overall variation rate determines the total number of variants on a haploid of the simulated individual genome. This parameter can be set as an interval.

**TABLE 2:** User-controlled parameters.

**Parameter**	**Type**	**Form**	**Range/examples**	**Default value**
Gender	String	Value	Male/female/any	Any
Overall variation rate	Numeric	Min, max	0 ≤ *OVR* ≤ 1	0.001
Known variation rate	Numeric	Min, max	0 ≤ *KVR* ≤ 1	0.9
Common variation rate	Numeric	Min, max	0 ≤ *CVR* ≤ 1	0.8
Background population	String	Value	AF, CAF, EUR_AF…	CAF
Number of novel coding variants	Numeric	Min, max	0 ≤ *CDN* ≤ 10,000	200
Novel Indel rate	Numeric	Min, max	0 ≤ *NIDR* ≤ 1	0.1
Maximum novel Indel length	Numeric	Value	2 ≤ *NIDL* ≤ 200	50
Ti/Tv ratio	Numeric	Min, max	1 ≤ *TiTv* ≤ 5	2.0, 2.1
Ti/Tv ratio in coding region	Numeric	Min, max	1 ≤ *TiTvC* ≤ 5	2.8, 3.0
Frame shifting rate	Numeric	Min, max	0 ≤ *FSR* ≤ 1	0.2, 0.3
Number of structural variations	Numeric	Min, max	0 ≤ *SVN* ≤ 1,000	0
Number of pathogenic variants	Numeric	Min, max	0 ≤ *PVN* ≤ 1,000	0

To ensure that the size of the generated personal genome variants meets user requirements, PGsim randomly selects or generates variants in different ways. According to the sources, the simulated variants can be divided into three types: known variants, novel variants, and special variants. Known variants are those that have already been detected from real individuals and already exist in known variant databases or common variant databases. Novel variants are randomly generated by PGsim. Special variants are variants from the SV database or disease-related variant database. Regarding authenticity, the number of special variants is much smaller than the known and novel variants, usually numbering up to a few hundred. If the variants from different sources conflict, PGsim preferentially incorporates special variants into the simulated personal genome, followed by known variants and finally the novel variants with the lowest priority.

PGsim employs different methods to simulate variants from the three sources. For known variants, we make a random sampling based on the corrected AF. For novel variants, we randomly generate a series of genome position coordinates based on user parameters, mutation laws, the reference genome sequence, and coding region data. For special variants, we randomly select the given number of records from the databases.

#### Known Variant Simulation

With the advancements of many large-scale genome sequencing projects and cohort sequencing, a fairly comprehensive understanding of human genome variation has been gained. In the real human individual genome, most variants are known variants, and most of the known variants are common variants. Therefore, the simulated personal genome should generally contain a considerable proportion of known and common variants, especially variants that fit the characteristics of the assumed population.

PGsim determines the proportion of known variants among all simulated variants through the known variation rate (KVR) parameter. The common variation rate (CVR) parameter determines the proportion of common variants among the known variants. The background population parameter determines which population’s AF is used by PGsim as a basis for randomly selecting common variants. This parameter should be set to the tag of the corresponding AF in the INFO field of the VCF file.

For each common variant in the database, PGsim performs random sampling once. When the random number is less than the AF of the given background population, the variant is selected as a genome variant of the simulated individual. To meet the user configuration parameters of the variant amounts such as OVR, KVR, and CVR, we calculated a correction factor, the practical AF coefficient, paC, through the input parameters and databases statistics,

$$\text{paC}=\frac{\mathrm{Requested}\,\mathrm{number}\,\mathrm{of}\,\mathrm{common}\,\mathrm{variants}}{\mathrm{Expected}\,\mathrm{number}\,\mathrm{of}\,\mathrm{common}\,\mathrm{variants}}=$$

(1) $$\frac{\text{GLN}\cdot \text{OVR}\cdot \text{KVR}\cdot \text{CVR}}{\text{ecvNum}}$$

where GLN denotes the total length of the reference genome, OVR denotes the overall variation rate, KVR denotes the known variation rate, CVR denotes the common variation rate, and ecvNum denotes the expected number of common variants, i.e., the sum of the non-reference allele frequencies of the common variants in the background population.

OVR, KVR, and CVR are user-controlled parameters. GLN, ecvNum, and paC are calculated by PGsim based on the user configuration and database statistics, namely, “internal parameters.” The internal parameter CVT (default CVT = 0.01) determines the minimum AF threshold of common variants. All internal parameters are illustrated in Table 3.

**TABLE 3:** Internal parameters.

**Parameter**	**Variable**	**Calculation method**
Threshold of allele frequency of common variants	*CVT*	Default *CVT* = 0.01
Power law ‘alpha’ of Indel length distribution	*PLalpha*	Default *PLalpha* = 1.8
Length of the reference genome	*GLN*	Scan reference genome
Number of ‘N’s in the reference genome	*nGLN*	Scan reference genome
Length of protein-coding regions	*modLen*	Scan gene model
Number of known variants in database	*varNum*	Scan known variants database
Number of common variants in database	*cvNum*	Scan common variants database
Expected total AF of common variants	*ecvNum*	Scan common variants database
Practical AF coefficient of common variants	*paC*	Eq. 1
Practical average AF of non-common variants	*paAF*	Eq. 2
Practical number of novel variants	*pnvNum*	Eq. 3
Expected overlap rate of known and novel variants	*eoR*	Eq. 4

**FIGURE 2:**
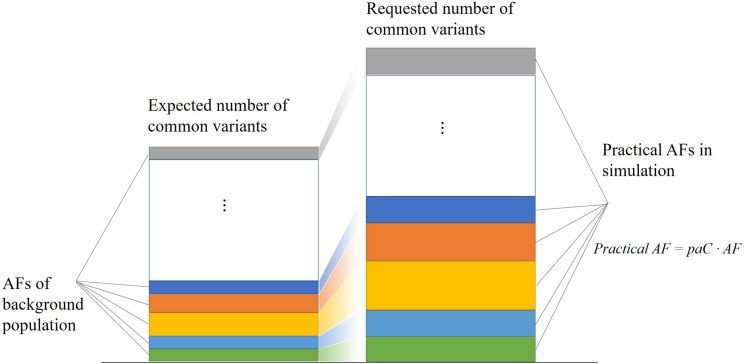
Selecting a specific number of common variants while preserving their relative occurrence probabilities.

The practical AF coefficient corrects the common variant allele frequencies of the background population in simulation. As shown in Figure 2, this strategy enables PGsim to select a specific number of common variants while preserving their relative probabilities of occurrence.

Non-common variants are randomly selected from the known variant database with the same probability, i.e., the practical average AF, or paAF. The internal parameter is also calculated based on user input parameters and databases statistics,

(2) $$\mathrm{paAF}=\frac{\text{GLN}\cdot \text{OVR}\cdot \mathrm{KVR}.\left(1-\mathrm{CVR}\right)}{\text{varNum}-\text{cvNum}}$$

where GLN denotes the total length of the reference genome, OVR denotes the overall variation rate, KVR denotes the known variation rate, CVR denotes the common variation rate, varNum denotes the number of records in the known variant database, and cvNum denotes the number of records in the common variant database. The internal parameters varNum and cvNum are obtained from databases statistics.

#### Novel Variant Simulation

Due to biochemical principles and selection pressure, the mutations that actually occur in the human genome are not completely random but follow certain rules, such as the Indel incidence and the Ti/Tv ratio. In the process of simulating an individual genome, these rules should be followed as closely as possible. Our method for simulating known variants basically retains the relative differences between allele frequencies and thus naturally follows the corresponding laws. However, the simulation of novel variants must carefully consider the influence of these factors.

The above rules are fairly different between protein-coding regions and non-coding regions. Therefore, PGsim simulates novel variants in coding and non-coding regions. Non-coding region variants are randomly selected from the while genome according to a uniform distribution. If the total number of non-coding region variants is pnvNum, then,

pnvNum=GLN⋅OVR⋅(1-KVR)⋅(1+eoR)⋅

(3) $$\frac{\text{GLN}}{\text{GLN}-\text{nGLN}-\text{modLen}}$$

where GLN denotes the total length of the reference genome, OVR denotes the overall variation rate, KVR denotes the known variation rate, nGLN denotes the length of “N”s in the reference genome, modLen denotes the length of the coding region, and eoR denotes the expected rate of novel variants overlapping with known variants. The internal parameter eoR is calculated by the following equation:

(4) $$\mathrm{eoR}=\frac{\text{OVR}\cdot \text{KVR}}{1-\text{OVR}\cdot \text{KVR}}$$

where OVR denotes the overall variation rate and KVR denotes the known variation rate.

pnvNum is greater than the number of novel variants set by the users through the OVR and KVR parameters. This is because during the process of merging simulated variants from different sources, novel variants have lower priority and may be discarded for a variety of reasons. The total number will be insufficient after discarding these novel variants. For example, some novel variants may conflict with the extracted known variants.

In addition, the coordinate positions of the novel variants are randomly generated according to a uniform distribution, which is basically suitable for non-coding regions. However, the distribution of variation between the coding and non-coding regions should obviously not be uniform. Therefore, PGsim allows users to set the number of novel variants in the coding region independently through the parameter CDN and then randomly generate the user configured number of novel variants in the coding region. Variants that fall into the coding region during the global novel variant simulation are discarded. Finally, novel variants that fall into the undetermined region of the genome, that is, regions where the reference genome sequence are “N”s, will also be considered invalid variants.

PGsim simulates both novel SNVs and novel Indels. The novel Indel rate (NIDR) parameter controls the proportion of novel Indels in all novel variants. NIDR applies to both coding and non-coding regions. However, for the coding regions, a portion of the frame shifting Indels may be discarded in the subsequent step.

PGsim allows the probability of the alternate allele selection of the novel SNVs to meet the user-controlled parameters Ti/Tv ratio (TiTv), and Ti/Tv ratio in the coding regions (TiTvC). The two parameters are applied in the non-coding regions and the coding regions, respectively.

**FIGURE 3:**
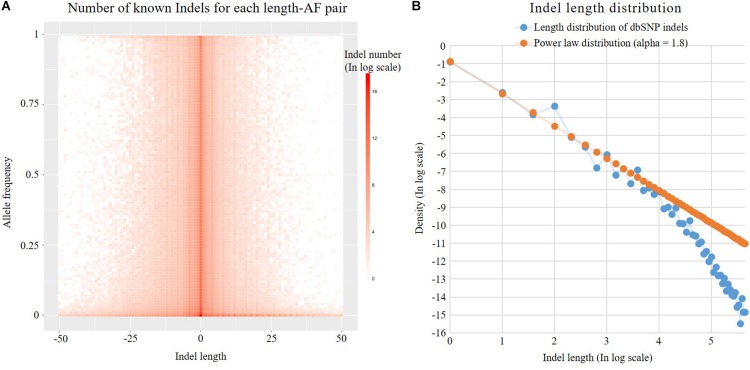
Indel distribution. **(A)** Number of known Indels for each length-AF pair. **(B)** Known dbSNP Indel length distribution and theoretical discrete power law distribution. Both axes are in log2 scale. The labels of the axes are the corresponding exponents.

Previous studies have shown that the Indel length distribution on the human genome follows a power law distribution (Cartwright, 2009). As shown in Figure 3, we analyzed the Indel length distribution in the dbSNP database. Figure 3A shows that the distribution of Indel length is basically consistent at different AF levels and is basically symmetrical between deletion (Indel length < 0) and insertion (Indel length > 0). Figure 3B shows that the known Indel length distribution in the database and the sampling results of a standard power law distribution with parameter alpha = 1.8 are basically the same. The distortion at the end of the figure may be due to the insufficient capability of long Indel detection by existing high-throughput sequencing technologies. The power law distribution is a long-tailed distribution. Statistics show that 99% of Indels in the dbSNP database are <20 nt in length. Here, we employ the following power law distribution to simulate the length of Indels:

(5) $$\mathrm{Indel}\,\mathrm{length}∼x^{\frac{1}{\left(1-\text{PLalpha}\right)}}$$

where PLalpha denotes the parameter alpha of the power law distribution. The default value of the internal parameter is 1.8.

The maximum length of novel Indels is determined by the user-controlled parameter “maximum novel Indel length” (NIDL). PGsim conducts random sampling from the discrete power law distribution to determine the length of each Indel. If the sampled value is larger than NIDL, the remainder of the division of the length by NIDL will be used as the length of the novel Indel.

For coding region Indels, the non-frameshifting Indels, i.e., novel Indels with a length of integer multiples of 3, are reserved. Only a portion of the remaining frameshifting Indels are reserved. The retention ratio is set by the frameshifting retaining rate (FSR) parameter.

#### Special Variant Simulation

All special variants are extracted from the databases. The user can set the number of special variants in the input parameters. The number should not exceed the total number of non-overlapping special variants in the database. For SV, PGsim first scans the SV database to group overlapped SVs, then randomly selects the given number of groups according to the SV number (SVN) parameter, and finally randomly selects a record in each group. For disease-related variants, PGsim directly extracts the given number of records from the database according to the pathogenic variants number (PVN) parameter. These randomly selected special variants are extracted during the planning phase of the personal genome simulation and subsequently incorporated into the generated VCF file by the order of the genome coordinates.

## Implementation

PGsim is implemented in Perl and can be run directly on MAC OS and Linux systems. PGsim consists of three components, each of which is an independent script. PG_planner.pl reads the parameter configuration file, scans the databases, and plans the individual genome simulation. PG_simulator.pl produces simulated individual genome variant data. PG_generator.pl generates diploid personal genome sequence data.

The user should specify the file paths of the reference genome and the variant databases in the configuration file. The reference genome data should be stored in FASTA format or its gzip-compressed file. The coding region information should be stored in a BED-format file. The variant databases should be stored in the standard VCF format files or their gzip-compressed files. The databases can be easily replaced on demand. The INFO field of the VCF file of the common variant database should contain AF information for the background population specified by the user.

To achieve better randomness, most of the input parameters can be set as an interval. During the planning phase of the simulation, these parameters will be randomly determined as a value within the interval according to the uniform distribution. Users can input equal upper and lower bounds for the interval to ensure that the parameter is set to a specific value.

PGsim is a single-threaded tool. However, if the databases are stored in gzipped form, extra threads for uncompressing the data in real time are needed by the gzip program. Multiple cores will accelerate the overall performance. During the whole process, PGsim generally does not occupy over 3 GB of memory, which is the size of the human reference genome.

The time of a single run of PGsim is mainly related to the size of the variant databases and less related to the number of simulated variants. This is because PGsim will traverse the variant database and conduct sampling for each known variant to ensure that all variants have a chance to be selected. According to repeated testing, if the entire dbSNP database is used as the known variants database and the common variants in dbSNP151 are used as the common variants database, then the database scanning time is ∼2 h, and the simulation time of the personal genome is ∼3 h. In this case, a total of 660,146,174 known variants are sampled, accounting for <20% of the whole genome length. If 37,302,978 common variants in the common SNP database are used for simulation, the database scanning time is approximately 18 min. Table 4 lists the typical time consumption of the major steps of PGsim in the individual genome simulation process. Each value is the average of 10 experiments.

**TABLE 4:** Average time consumption of PGsim.

**Operation**	**Major factor influencing performance**	**Value**	**Average time (s)**
Reference genome scanning and analysis	Length	3,088,286,401	109
Coding region scanning and analysis	Records	211,255	2
Known variant database scanning and analysis	Records	660,146,174	7,299
Known variant database scanning and analysis	Records	37,302,978	305
Common variant database scanning and analysis	Records	37,302,978	1,045
Novel variants planning	pnvNum	∼300,000	4
Variant simulation	Known DB records	660,146,174	10,702
Variant simulation	Known DB records	37,302,978	2,275
Personal genome sequence generation	Genome length	3,088,286,401	286

The database scanning process will only be performed when the database is used for the first time. After scanning, PG_planner will generate a statistical results file with the database file name as the prefix and “pgstat” as the extension. When used again, PG_planner detects whether this file exists. If so, it will directly read the statistical results.

## Discussion

In this paper, we designed and developed a comprehensive and highly customizable individual genome simulator, PGsim. The majority of variants in human genomes are known variants, and most of the known variants are common variants. The AF of common variants varies among individuals of different populations. PGsim can make full use of the real AF information in the known variant database to simulate a personal genome whose background population characteristics conform to the user’s requirements.

Users can flexibly control the proportion and quantity of known variants, common variants, novel variants in both coding and non-coding regions, and special variants through detailed parameter settings. Our method leverages the existing knowledge of genomics and population genetics, such as the Ti/Tv ratio, Indel incidence, and Indel length distribution, to simulate individual genomes that are consistent with biochemical principles and selection processes. To ensure that the simulated personal genome has sufficient randomness, PGsim makes the generated variants more real and reliable in terms of variant distribution, proportion, and population characteristics.

PGsim is able to employ a very large-volume database as background data to simulate personal genomes and does not require SQL database support. Users can easily change the variant databases used as needed. PGsim traverses the gzip-compressed database file to minimize the memory and hard disk usage in the personal genome simulation process. As a Perl script, there is no obstacle to running PGsim on any version of the MAC OS or Linux systems, and no libraries, packages, interpreters, compilers, or other dependencies need to be installed in advance.

## Data Availability Statement

The PGsim tool is available at https://github.com/lrjuan/PGsim. An example configuration file and GRCh38/hg38 CDS data in BED format are also provided. Other required reference sequences and variant databases can be downloaded from the NCBI FTP site. A README file is uploaded with the scripts. Users can also use “-help” or “-h” parameter to check the program usage.

## Author Contributions

LJ and YDW designed the study. LJ, YTW, JJ, and QY developed the tool. LJ and QJ wrote the manuscript.

## Conflict of Interest

The authors declare that the research was conducted in the absence of any commercial or financial relationships that could be construed as a potential conflict of interest.
